# Overview of OVATE FAMILY PROTEINS, A Novel Class of Plant-Specific Growth Regulators

**DOI:** 10.3389/fpls.2016.00417

**Published:** 2016-03-31

**Authors:** Shucai Wang, Ying Chang, Brian Ellis

**Affiliations:** ^1^Key Laboratory of Molecular Epigenetics of MOE and Institute of Genetics and Cytology, Northeast Normal UniversityChangchun, China; ^2^College of Life Science, Northeast Agricultural UniversityHarbin, China; ^3^Michael Smith Laboratories, The University of British Columbia, VancouverBC, Canada

**Keywords:** OVATE, OVATE FAMILY PROTEINS, fruit shape, transcription factor, plant growth and development, *Arabidopsis*, rice, pepper

## Abstract

OVATE FAMILY PROTEINS (OFPs) are a class of proteins with a conserved OVATE domain. OVATE protein was first identified in tomato as a key regulator of fruit shape. OFPs are plant-specific proteins that are widely distributed in the plant kingdom including mosses and lycophytes. Transcriptional activity analysis of *Arabidopsis* OFPs (AtOFPs) in protoplasts suggests that they act as transcription repressors. Functional characterization of OFPs from different plant species including *Arabidopsis*, rice, tomato, pepper, and banana suggests that OFPs regulate multiple aspects of plant growth and development, which is likely achieved by interacting with different types of transcription factors including the KNOX and BELL classes, and/or directly regulating the expression of target genes such as *Gibberellin 20 oxidase* (*GA20ox*). Here, we examine how OVATE was originally identified, summarize recent progress in elucidation of the roles of OFPs in regulating plant growth and development, and describe possible mechanisms underpinning this regulation. Finally, we review potential new research directions that could shed additional light on the functional biology of OFPs in plants.

## Introduction

More than 100 years ago, it was proposed that the pear-shaped fruit phenotype in tomato (*Solanum lycopersicum*) might be genetically controlled by a single recessive locus, namely *ovate* (Hedrick and Booth, 1907; Price and Drinkard, 1908). However, it was only at the end of the last century that Ku et al. (1999) established, by construction and analysis of near-isogenic lines (NILs), that the *ovate* locus could account for both the pear shape and the elongated fruit shape in tomato. The *ovate* locus was later mapped to chromosome 2 (Ku et al., 2001), and the *ovate* gene was finally cloned in 2002 (Liu et al., 2002). Amino acid sequence analysis showed that OVATE is different from all the previously characterized plant genetic regulators, indicating that OVATE FAMILY PROTEINS (OFPs) represent a novel class of plant regulators (Liu et al., 2002).

Subsequent studies revealed that OFPs are widely distributed in the plant kingdom, and that they regulate multiple aspects of plant growth and development (Gui and Wang, 2007; Rodríguez et al., 2011; Tsaballa et al., 2011; Wang et al., 2011; Huang et al., 2013).

## The 100-Year Pathway to the Identification of the *OVATE* Gene

About a century ago, a series of genetic studies in tomato suggested that fruit size and shape in this economically important species are quantitatively inherited (Hedrick and Booth, 1907; Price and Drinkard, 1908; Grane, 1915; Lindstrom, 1926, 1927, 1929, 1932; MacArthur, 1926; Tanksley, 2004). More specifically, pear-shaped fruit form in tomato was proposed to be controlled by a single recessive quantitative trait locus (QTL), which was named *pyriform* (*pr*) (Hedrick and Booth, 1907; Price and Drinkard, 1908). Later, *pr* was found to co-segregate with the locus causing oblate- to oval-shaped fruit, and was therefore renamed *ovate* (*o*). In the late 1920s, based on its linkage to the *dwarf* locus, *ovate* was placed on chromosome 2 (Lindstrom, 1926, 1927, 1929; MacArthur, 1926).

Nearly 70 years later, Ku et al. (1999) conducted a more detailed molecular marker-based analysis of fruit shape in *Lycopersicon*. By crossing *Lycopersicon esculentum* cv. Yellow Pear (TA503), a variety of tomato bearing small, pear-shaped fruit, with *Lycopersicon esculentum pimpinellifolium* (LA1589), a wild tomato species bearing round-shaped fruit, and examining the F2 population, they found that the pear-shaped fruit phenotype is largely controlled by a major QTL on chromosome 2. This observation was confirmed by analyzing F2 populations from crosses between TA503 and a round-fruited introgression tomato line IL2-5, which carried the distal portion of the chromosome 2 from the *L. pennellii* genome. Based on these results, they proposed that the QTL detected on chromosome 2 corresponds to the *ovate* locus (Ku et al., 1999).

High-resolution mapping of the *ovate* region on chromosome 2 using a total of 3000 near-isogenic lines (NILs) derived from TA503 and *L. pennellii*, allowed Ku et al. (2001) to place *ovate* adjacent to a known marker. By screening tomato BAC (bacterial artificial chromosome) and binary BAC libraries with the known marker-derived probe, and mapping the ends of the selected BAC clones, they were able to identify two *ovate*-containing BAC clones (Ku et al., 2001). A combination of sequencing and fine mapping analysis of one of these BAC clones obtained further narrowed the *ovate* locus to a 55 kb fragment that contained eight open reading frames (ORFs) (Liu et al., 2002). To identify the *OVATE* gene, Liu et al. (2002) amplified and compared the corresponding 55 kb fragments from TA496, a round-fruited wild type *L. esculentum* genotype, and TA493 (*L. esculentum* cv. Heinz 1706), an *ovate* genotype. They identified a G_TA496_-to-T_TA493_ nucleotide polymorphism (SNP) in one of ORFs. This SNP created an early stop codon in the *ovate* genotype, leading to a 75-aa truncation in the C-terminus of the predicted protein. Sequence comparison of the corresponding ORF in several pear-shaped tomato varieties including TA503, LA791 (*L. esculentum* cv. Longjohn), and LA0025, as well as complementation of the pear-shaped fruit phenotype in TA503 by over-expression of the genomic DNA fragment containing the ORF and its 5′ and 3′ untranslated regions from TA496, confirmed the identity of the tomato *OVATE* gene.

Amino acid sequence analysis showed that the OVATE protein contains an ~70-aa carboxyl-terminal domain, referred to as the OVATE domain, that is conserved in both *Arabidopsis* and rice. The premature termination occurring in the *ovate* genotype eliminates most of this conserved OVATE domain (Liu et al., 2002). The OVATE protein also contains a putative bipartite nuclear localization signal (NLS), and two putative Von Willebrand factor type C (VWFC) domains required for protein–protein interaction, features that distinguish OVATE from any of the previously identified plant genetic regulators (Liu et al., 2002).

Thanks to fast development of new sequencing technologies over the recent years, the genomes of many more plant species have now been fully sequenced, which has greatly facilitated the identification of protein homologs and phylogenetic studies in plants. Based on amino acid sequence similarity analysis, OFPs are found exclusively in plants (Hackbusch et al., 2005; Gui and Wang, 2007; Wang et al., 2007, 2011; Rodríguez et al., 2011; Tsaballa et al., 2011; Huang et al., 2013). By using the amino acid sequences of OFPs in *Arabidopsis* and the OVATE protein in tomato to search genomes of 13 land plants including *Solanum lycopersicum*, *Solanum tuberosum*, *Mimulus guttatus*, *Arabidopsis*, *Vitis vinifera*, *Populus trichocarpa*, *Prunus persica*, *Carica papaya*, *Aquilegia coerulea*, rice (*Oryza sativa*), *Zea mays*, *Selaginella moellendorffii*, and *Physcomitrella patens*, Liu et al. (2014) found that OFPs are distributed in all the plants examined, including the seedless vascular plant *Selaginella moellendorffii*, and the non-vascular plant *Physcomitrella patens*. In contrast, no OFPs were identified in chlorophytes, a division of the green algae (Liu et al., 2014). Interestingly, monocot species appeared to have more OFP family members than eudicots; for example, *Zea mays* had 45 OFPs, and rice had 33 OFPs, whereas tomato had 26 OFPs and *Vitis vinifera* had only 9 (Liu et al., 2014). It was subsequently reported that 31 genes in rice encode full-length OFPs (Yu et al., 2015). It should also be noted that Liu et al. (2014) found that the *Arabidopsis* genome contains 19 OFP-encoding genes, rather than 18 as reported previously (Hackbusch et al., 2005; Wang et al., 2011).

## Roles of OFPs in Plant Growth and Development

Although OFPs are widely distributed in the plant kingdoms, their biological functions in plants remain largely unknown. However, limited studies in several different plant species including tomato, *Arabidopsis*, pepper (*Capsicum annuum*) and banana (*Musa acuminata*) have revealed that OFPs proteins are involved in regulation of several aspects of plant growth and development.

### Ovule Development

Characterization of a T-DNA insertion mutant for AtOFP5 suggests that this member of the *Arabidopsis* OFP family may be involved in the regulation of ovule development. When Pagnussat et al. (2007) failed to isolate homozygous plants from a T-DNA insertion line of *AtOFP5* (SALK_ 010386), they examined female gametophyte development in pistils of the *ofp5-/+* heterozygotes. This revealed that, out of the 256 embryo sacs studied, ~38% of them seemed to collapse at the FG2 female gametophyte development stage, when two-nucleate cells are normally produced, and ~8% of the embryo sacs showed abnormal micropylar cells with two egg cells, indicating that *AtOFP5* is required for proper female gametophyte development. Because some embryo sacs in the *ofp5-* genetic background had two egg cells, AtOFP5 may be specifically involved in the regulation of a cell fate switch from synergid to egg cell development (Pagnussat et al., 2007).

### Vascular Development

*OsOFP2*, a rice OFP gene, was found to be expressed mainly within the vasculature in all growth stages examined in rice, as shown by GUS staining in *OsOFP2pro:GUS* transgenic rice plants. Rice plants over-expressing *OsOFP2* under the control of the *35S* promoter showed reduced height and exhibited altered leaf morphology, seed shape, and positioning of vascular bundles in the stems. Transcriptome analysis of the *OsOFP2* over-expressing rice plants showed that the expression of genes associated with vascular development, lignin biosynthesis, and hormone homeostasis was altered, consistent with a role for *OsOFP2* in the regulation of vascular development (Schmitz et al., 2015).

### Fruit Shape

Consistent with OVATE’s role as a key regulator of fruit shape in tomato (Liu et al., 2002; Azzi et al., 2015; Wang et al., 2015; Wu et al., 2015), the *OVATE* gene is expressed mainly in reproductive organs. Its transcripts can be detected in tomato flowers 10 days before anthesis and begin to decrease in developing fruit 8 days after anthesis (Liu et al., 2002). *CaOvate*, an *OVATE* family member in pepper that shares 63% identity with the tomato OVATE protein, was also found to be involved in the regulation of fruit shape (Tsaballa et al., 2011). However, unlike in tomato, differences in fruit shape in two pepper cultivars, cv. “Mytilini Round” and cv. “Piperaki Long,” are associated with different patterns of *CaOvate* expression, rather than with an ORF mutation; i.e., the expression of *CaOvate* is higher in cv. “Mytilini Round” than in cv. “Piperaki Long.” Down-regulation of *CaOvate* in cv. “ Mytilini Round” produced through virus-induced gene silencing (VIGS) thus changed its fruit to a more oblong form (Tsaballa et al., 2011). Beyond the Solanaceae, several QTLs controlling fruit shape in melon (*Cucumis melo*) have also been identified, and several melon OFP homologs (*CmOFP*) were found to co-map with seven fruit shape QTLs, indicating that OFPs in melon may also control fruit shape (Monforte et al., 2014).

It should be noted that the G-to-T mutation in *OVATE* is not associated with a single fruit shape phenotype in tomato. In some genetic backgrounds, the mutation leads to pear-shape fruits, but in other backgrounds, fruit shape remains largely unchanged, indicating that the *OVATE* locus may interact with other regulators to control a specific fruit shape (Tanksley, 2004; Gonzalo and van der Knaap, 2008). Indeed, two suppressor loci for *OVATE*, *suppressor of OVATE1* (*sov1*) and *sov2*, have been identified in tomato (Rodríguez et al., 2013).

Grafting of *Capsicum* cv. “Mytilini Round” scion on the long-shaped cultivar, cv. “Piperaki Long” rootstock, also resulted in heritable fruit shape changes in the scion, and these fruit phenotypic changes were retained through two generations of seed-derived progeny (Tsaballa et al., 2013). However, only a slight difference in *CaOvate* gene expression accompanied this change in the fruit shape, and simple sequence repeat (ISSR) analysis of the progenies of the scion fruits showed that their genetic profile closely resembled the parental scion genetic profile. This suggests either that only slight changes in the expression of *CaOvate* are enough to induce fruit shape changes, or that *CaOvate* interacts with other genes in an epistatic manner to regulate fruit shape in pepper (Tsaballa et al., 2013). It is also possible that epigenetic modification plays a role in the regulation of fruit shape in pepper.

### DNA Repair

When Hackbusch et al. (2005) tried to characterize *Arabidopsis ofp1* mutants, they failed to recover any homozygous plants from three independent lines with T-DNA inserted either in the single exon or in the 5′ upstream region of *AtOFP1*. They concluded that *AtOFP1* must be required for essential processes in gametophyte or sporophyte development. However, they did not observe any apparent morphological aberrations in either the pollen or ovules in plants heterozygous for the T-DNA insertion. When they further investigated male and female transmission of the T-DNA insertion by reciprocally crossing the mutants with wild type plants, they found that when the heterozygous mutant lines were used as pollen donors, no T-DNA insertion in the *AtOFP1* gene was detected in the F1 plants. The authors therefore concluded that AtOFP1 is essential for male gamete and pollen function (Hackbusch et al., 2005).

In contrast to this report, Wang et al. (2007) successfully obtained homozygous mutant plants from *ofp1-2* and *ofp1-3*, two mutant lines derived from the same T-DNA insertion lines used by Hackbusch et al. (2005). These authors reported that, in the *ofp1-2* mutant, the T-DNA was actually inserted in the 5′-UTR of *AtOFP1* rather than in the exon as indicated by the T-DNA Express database (http://signal.salk.edu/cgi-bin/tdnaexpress). The expression of *AtOFP1* in the *ofp1-2* mutant was largely unaffected. Wang et al. (2007) also identified an *ofp1-1* mutant, a true loss-of-function mutant line for *AtOFP1* derived from a transposon insertion event, and found that this mutant is morphologically similar to the wild type plant. These results are inconsistent with the earlier proposal that *OFP1* plays a critical role in male gamete transmission or pollen function.

Although the *ofp1-1* mutant is largely similar to wild type plant (Wang et al., 2007), *ofp1-1* mutant seedlings were reported to be more sensitive to treatment with methyl methanesulfonate (MMS), a DNA-damaging reagent. The *ofp1-1* mutants also showed relative lower non-homologous end-joining (NHEJ) activity *in vivo*. Because the NHEJ pathway is thought to be involved in the repair of DNA double-strand breaks (DSBs), these results suggest that OFP1 may play a role in this DNA repair pathway (Wang et al., 2010).

### Secondary Cell Wall Formation

Although all the single and double *AtOFP* gene mutants identified in *Arabidopsis* are morphologically similar to wild type plants (Wang et al., 2011), careful examination of the anatomy of stem inflorescence cross-sections showed that *ofp4* mutants exhibited an *irregular xylem* (*irx*) phenotype, marked by increased thickness of interfascicular fiber cell walls, and decreased wall thickness of vessel and xylary fiber cells (Li et al., 2011). This phenotype is similar to that of *knat7*, a loss-of-function mutant of the *KNOTTED ARABIDOPSIS THALIANA7* transcription factor gene (Brown et al., 2005). Further analysis showed that the phenotype of the *ofp4 knat7* double mutant was similar to that of the *ofp4* or *knat7* single mutants, and that the *AtOFP4* over-expression phenotype (kidney-shaped cotyledons, and round and curled leaves) (Wang et al., 2011), was suppressed in a *knat7* mutant background. Taken together, these results suggest that AtOFP4 is involved in the regulation of secondary cell wall formation, and that its function is at least partially dependent on *KNAT7*.

Unlike *ofp4*, xylem and interfascicular fiber morphology in the *ofp1-1* mutant is indistinguishable from that in wild type plants (Li et al., 2011). However, the *AtOFP1* over-expression phenotype is similar to that of *AtOFP4* over-expression plants (Wang et al., 2011), and is also suppressed in a *knat7* mutant background, suggesting that AtOFP1 may also be involved in the regulation of secondary cell wall formation (Li et al., 2011). However, the possibility cannot be ruled out that this behavior may simply reflect the close homologous relationship between AtOFP1 and AtOFP4.

### Fruit Ripening

A role for OFPs in regulating fruit ripening has so far only been reported for banana. The banana OFP1 (MaOFP1) was identified in a yeast two-hybrid (Y2H) study when using the banana MADS-box protein MuMADS1 as bait to screen a 2-day-postharvest (DPH) banana fruit cDNA library (Liu et al., 2015). Quantitative RT-PCR (qRT-PCR) analysis showed that both *MuMADS1* and *MaOFP1* were highly expressed in 0 DPH fruit, but had relative low levels of expression in the stem. However, different expression patterns of *MuMADS1* and *MaOFP1* were observed in different tissues and developing fruits. qRT-PCR analysis also showed that expression of *MuMADS1* and *MaOFP1*was differentially regulated by ethylene, i.e., expression of *MuMADS1* was induced by exogenously applied ethylene, and suppressed by the ethylene competitor 1-methylcyclopropene (1-MCP), whereas expression of *MaOFP1* was suppressed by ethylene, and induced by 1MCP. These results indicate that MuMADS1 and MaOFP1 may play antagonistic roles in ethylene-induced postharvest fruit ripening in banana (Liu et al., 2015).

### Pleiotropic Effects

Although some evidence suggests that OFPs may have specific effects on different aspects of plant growth and development, as described above, other evidence suggests that OFPs can also have complex pleiotropic effects on plant growth and development. For example, in addition to resulting in round fruit, over-expression of *OVATE* in tomato also produced a number of abnormal phenotypes, including reduced size of floral organs, dwarf plants with smaller compound-leaf size and rounder leaflets (Liu et al., 2002). Similarly, over-expression of some *Arabidopsis OFP* genes also resulted in inhibited plant growth and development (Hackbusch et al., 2005; Wang et al., 2011).

**FIGURE 1:**
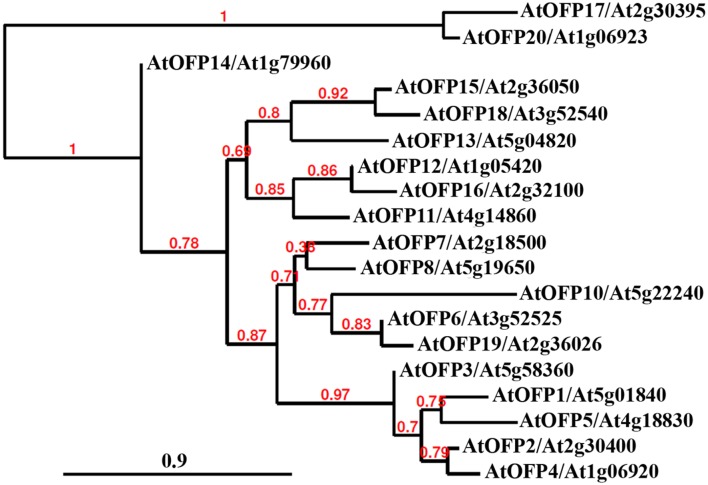
**Phylogenetic analysis of *Arabidopsis* OVATE FAMILY PROTEINS (AtOFPs).** The entire amino acid sequences of AtOFPs were obtained from Phytozome (https://phytozome.jgi.doe.gov/pz/portal.html) and used to generate the phylogenetic tree by using “One Click” mode with default settings on Phylogeny (www.phylogeny.fr). Branch support values are indicated above the branches, and Bar indicates branch length.

Analysis of transgenic plants over-expressing *AtOFPs* in *Arabidopsis* also reveals that they regulate multiple aspects of plant growth and development in this species. Based on the similarity of the phenotypes observed, AtOFPs could be grouped into different groups. Over-expression of *AtOFP1*, *AtOFP2*, *AtOFP4*, *AtOFP5* and *AtOFP7* resulted in similar phenotypes including kidney-shaped cotyledons, as well as round and curled leaves, and these AtOFPs were designated as Class I AtOFPs. Over-expression of *AtOFP6* and *AtOFP8*, on the other hand, resulted in a different phenotype including flat, thick and cyan leaves, and these were therefore designated as Class II AtOFPs. Over-expression of *AtOFP13*, *AtOFP15*, *AtOFP16* and *AtOFP18* led to another distinct phenotype including blunt-end siliques, and these AtOFPs were designated as Class III AtOFPs. Plants over-expressing all other AtOFPs examined were largely indistinguishable from wild type plants (Wang et al., 2011). Interestingly, this functional-based classification is largely consistent with the subgroups of the AtOFPs identified in phylogenetic analysis (**Figure 1**). Because *AtOFP19* and *AtOFP20* are newly identified OFPs in *Arabidopsis* (Liu et al., 2014), their functions have not yet been examined. Consistent with their functions in regulating multiple aspects of plant growth and development, expression of most of the *OFPs* in tomato, rice and *Arabidopsis* was detectable in all the tissues and organs examined (Wang et al., 2011; Liu et al., 2014; Schmitz et al., 2015). Nevertheless, differential expression patterns are also observed; for example, nearly half of the *OsOFPs* were found to be more highly expressed during the stages of rice seed development (Yu et al., 2015).

It is interesting that all the AtOFPs shown through genetic manipulations, as described above, to have a specific role in regulating plant growth and development are from the Class I subfamily. All knockout mutants identified so far for other *AtOFP* genes, including *AtOFP8*, *AtOFP10*, *AtOFP15*, and *AtOFP16*, are morphologically similar to wild type plants (Wang et al., 2011). A double mutant between two class III OFP genes, *ofp15 ofp16* is also indistinguishable from wild type plants (Wang et al., 2011).

## Mechanisms Underlying the Regulation of Plant Growth and Development By OFPs

### Regulation of Target Gene Expression

The evidence available so far supports a model in which OFPs regulate plant growth and development by directly influencing expression of their target genes, and/or through interaction with other transcription factors. Both AtOFP1 and AtOFP5 were found to associate with the cytoskeleton in transient expression assays in tobacco leaves (Hackbusch et al., 2005). However, similar to the tomato OVATE protein, AtOFP1 also possesses a putative NLS (Liu et al., 2002; Wang et al., 2007), and in both transient transfection assays in *Arabidopsis* protoplasts, and stable transformed *Arabidopsis* plants, AtOFP1 was found to localize in the nucleus (Wang et al., 2007). AtOFP1 also contains an LxLxL motif (Wang et al., 2007), which is also found in Aux/IAA proteins and ERF transcription factors and is required for their transcriptional repression functions (Ohta et al., 2001; Hiratsu et al., 2003; Tiwari et al., 2004). Transfection assays in *Arabidopsis* protoplasts showed that AtOFP1 repressed reporter gene expression when recruited to the promoter region of the *Gal4:GUS* reporter gene by a fused Gal4 DNA binding domain (GD), suggesting that AtOFP1 may function *in vivo* as a transcriptional repressor (Wang et al., 2007). Although some of the AtOFPs do not contain a LxLxL motif, all the AtOFPs examined repressed *Gal4:GUS* reporter gene expression in transfected protoplasts, indicating that AtOFPs in *Arabidopsis* are a novel transcription repressor family (Wang et al., 2011).

### Targets of OFP Proteins

A gain-of-function mutant of AtOFP1, *ofp1-1D*, as well as *AtOFP1* over-expressing plants, all showed reduced lengths in their aerial organs, including hypocotyl, rosette leaves, cauline leaves, inflorescence stem, floral organs and siliques (Hackbusch et al., 2005; Wang et al., 2007). Detailed analysis showed that this growth phenotype was the result of a reduction in cell elongation, rather than in cell division (Wang et al., 2007), suggesting that AtOFP1 represses cell elongation. Hackbusch et al. (2005) had earlier shown that expression of *AtGA20ox1*, a gene encoding a key enzyme in gibberellic acid (GA) biosynthesis, was reduced in plants over-expressing AtOFP1, and Wang et al. (2007) showed, by use of chromatin immunoprecipitation assays, that the *AtGA20ox1* promoter is a direct target of AtOFP1.

*GA20ox* is likely also a target of OFPs in pepper and rice. In pepper, two lines of evidence suggest that *GA20ox1* is likely a target gene of the OFP homolog, CaOvate. First, RT-PCR analysis showed that although *GA20ox1* has a similar expression level at most growth stages in both cv. “Mytilini Round” and cv. “Piperaki Long,” its expression was significantly higher in cv. “Piperaki Long” fruits 10 days after anthesis. In addition, expression of *GA20ox1* was elevated after VIGS suppression of *CaOvate* expression (Tsaballa et al., 2011, 2012). Similarly, rice *GA20ox7* is likely a target gene of OsOFP2, since expression of *GA20ox7* was reduced in transgenic rice plants over-expressing *OsOFP2* (Schmitz et al., 2015).

Although *GA20ox1* is probably a direct target of AtOFP1 (Wang et al., 2007), and reduction in the expression of rice *GA20ox7* is correlated with lower gibberellin content in transgenic rice plants (Schmitz et al., 2015), exogenous GA can only partially restore the defects in cell elongation in *Arabidopsis* transgenic plants over-expressing AtOFP1 (Wang et al., 2007). This indicates that AtOFP1 may have other target genes, and indeed, microarray-based gene expression assays showed that a total of 129 genes were down-regulated at least twofold when *AtOFP1-GR* (glucocorticoid receptor) transgenic plants were treated with DEX (dexamethasone), which will allow the AtOFP1-GR protein to relocate into the nucleus (Wang et al., 2011). However, it remains unknown if any of these genes is directly targeted by AtOFP1.

### Interaction of OFPs With Other Transcription Factors

So far nearly all OFPs with known functions were found to regulate plant growth and development via interaction with homeodomain proteins (**Table 1**). In a yeast two-hybridization screen, nine AtOFPs were found to interact with the 3-amino acid loop extension homeodomain (TALE) transcription factors KNOX and BELL (BEL1-like homeodomain) (Hackbusch et al., 2005). Consistent with the observation that the *ofp4 knat7* double mutant phenotype was similar to that of the *ofp4* or *knat7* single mutants, and that the *AtOFP1* and *AtOFP4* over-expression phenotype was suppressed in a *knat7* mutant background, transfection assays in *Arabidopsis* protoplasts showed that both AtOFP1 and AtOFP4 physically interacted with KNAT7 (Li et al., 2011). Transfection assays in *Arabidopsis* protoplasts also showed both OFP1 and OFP4 interacted with BLH6 (Liu and Douglas, 2015). Considering that MYB75/PAP1 (PRODUCTION OF ANTHOCYANIN PIGMENT 1), a R2R3 MYB transcription factor that has been shown to regulate phenylpropanoid biosynthesis in *Arabidopsis* (Borevitz et al., 2000), interacts with KNAT7 to modulate secondary cell wall deposition in stems and seed coat in *Arabidopsis* (Bhargava et al., 2013), it is likely that AtOFPs, MYB transcription factors, and TALE homeodomain proteins form one or more multi-protein complexes to regulate secondary cell wall formation. Recently, AtOFP1 was also found to interact with the BELL transcription factor BLH3 to regulate the vegetative to reproductive phase transition in *Arabidopsis* (Zhang et al., 2016).

**Table 1:** OVATE FAMILY PROTEINS (OFPs) interact with homeodomain proteins to regulate plant growth and development.

OFPs	Interactors	Functions in plants	Reference
AtOFP1	KNAT7, BLH6	Secondary cell wall formation	Li et al., 2011; Liu and Douglas, 2015
AtOFP1	BLH3	Phase transition	Zhang et al., 2016
AtOFP4	KNAT7, BLH6	Secondary cell wall formation	Li et al., 2011; Liu and Douglas, 2015
AtOFP5	KANT3, BLH1	Female gametophyte development	Pagnussat et al., 2007
GhOFP4	GhKNL1	Secondary cell wall formation	Gong et al., 2014
OsOFP2	OsKNAT7, BLH6-like1, bHLH6-like2	Vascular development	Schmitz et al., 2015
MaOFP1	MuMADS1	Fruit ripening	Liu et al., 2015

Homologs of *Arabidopsis* KNOX and BELL proteins have also been fund to interact with OFPs and to regulate secondary cell wall formation in other plant species. For example, GhKNL1, a homeodomain protein in cotton (*Gossypium hirsutum*) is found to be preferentially expressed in developing fibers at the stage of secondary cell wall biosynthesis, and ectopic expression of *GhKNL1* can partially rescue the cell wall defective phenotype of the *Arabidopsis knat7* mutant. Yeast two-hybrid assays showed that GhKNL1 interacts with GhOFP4, as well as with AtOFP1, AtOFP4, and AtMYB75 (Gong et al., 2014). In rice, OsOFP2 was found to interact with putative vascular development KNOX and BELL proteins, so it is also likely that OsOFP2 can modulate KNOX-BELL function in this species to regulate diverse aspects of development, including vascular development (Schmitz et al., 2015).

*AtOFP5* has been shown to be involved in the regulation of female gametophyte development. The “two egg cells” phenotype in *eostre-1*, a female gametophyte mutant, was caused by elevated expression of the BELL transcription factor gene *BLH1* in the embryo sac, and this phenotype is dependent upon the function of the class II knox gene, *KNAT3*, (Pagnussat et al., 2007). Disruption of AtOFP5, a known interactor of KNAT3 and BLH1, also partially phenocopies the *eostre* mutation (Pagnussat et al., 2007), suggesting that the roles of *AtOFP5* in female gametophyte development may also involve interactions of AtOFP5 with TALE homeodomain proteins.

On the other hand, Y2H screening using tomato OVATE protein as bait did not lead to the identification of any KNOX or BELL transcription factors. Instead, the screen revealed interactions of OVATE with 11 out of 26 members of the TONNEAU1 Recruiting Motif (TRM) superfamily, including the putative tomato ortholog of AtTRM17/20 (van der Knaap et al., 2014). Because some of the TRMs can bind microtubules and are likely centrosomal components in plant cells (Drevensek et al., 2012), this result suggests that, in addition to interacting with KNOX or BELL transcription factors, and acting as transcriptional repressors to repress target gene expression, OFPs may also interact with TRMs and microtubules to regulate plant growth and development (van der Knaap et al., 2014).

## Concluding Remarks and Future Perspectives

Studies in recent years reveal that OFPs are widely distributed in the plant kingdom, and that they help regulate multiple aspects of plant growth and development in various species (Liu et al., 2002; Hackbusch et al., 2005; Gui and Wang, 2007; Wang et al., 2007, 2011; Rodríguez et al., 2011; Tsaballa et al., 2011; Huang et al., 2013). Beyond this general observation, however, it will be important to establish the extent to which OFP family members are functionally conserved in different taxa. As described above, it would appear that some OFP homologs may have conserved functions; for example, OVATE protein was identified as a key regulator of fruit shape in tomato (Liu et al., 2002), while fruit shape in pepper is also at least partially controlled by an OFP homologue, CaOvate (Tsaballa et al., 2011). However, tomato and *Capsicum* are closely related genera, so it remains to be established whether fruit shape in other plants is also controlled by homologous OFPs. In addition, a major QTL, *fs10.1*, has been shown to control fruit elongation in *Capsicum*, but it is unclear if it represents an *OFP* gene (Borovsky and Paran, 2011). Similarly, several QTLs controlling fruit shape in apple have been identified, but so far it is unclear whether any of them is associated with an *OFP* gene (Cao et al., 2015). Once these genomes have been fully sequenced, the candidate regions can be explored for the possible presence of OFP homologs.

Further evidence is also required to establish the extent to which the function of OFPs in controlling secondary cell formation might be evolutionarily conserved. In *Arabidopsis*, interaction of specific OFPs with homeodomain proteins strongly influences secondary cell wall formation (Li et al., 2011; Liu and Douglas, 2015). In cotton (*Gossypium hirsutum*), GhKNL1 has been reported to interact with GhOFP4, but it remains to be demonstrated that GhOFP4 is also involved in the regulation of secondary cell wall formation in cotton fibers.

So far only *AtGA20ox1* has been shown to be a direct target of AtOFP1. However, the observation that expression of >100 genes was down-regulated in DEX-treated *AtOFP1-GR* transgenic plants (Wang et al., 2011), raises the possibility that some of these genes may also serve as direct targets of AtOFP1.

The possibility cannot be excluded that OFPs may regulate plant growth and development in ways other than directly targeting expression of particular genes or interaction with other transcription factors. Phenotype similarity-based characterization may make it possible to detect the operation of a previously unsuspected mechanism. For example, transgenic *Arabidopsis* over-expressing Class III AtOFP genes display blunt siliques, a phenotype also observed in the mutants *erecta*, a loss-of-function mutant of *ERECTA* (*ER*), a putative receptor protein kinase, and *agb1-1*, a loss-of-function mutant of the heterotrimeric G-protein β subunit gene *AGB1* (Torii et al., 1996; Lease et al., 2001). The *agb1-1* mutant was originally identified as an *erecta-like* mutant, *elk4*, during the characterization of an ER signaling pathway, and was renamed as *agb1-1* after *ELK4* was found to encode AGB1. Their results indicate that AGB1 can influence silique morphology via an ER-type Leucine-rich repeat (LRR) receptor-like kinase signaling pathway. Considering that an ER LRR receptor-like kinase signaling pathway has been shown to regulate multiple aspects of plant growth and development, including organ shape (Shpak et al., 2003), and that the transcription factor SPEECHLESS has been shown to be phosphorylated by a MAP kinase signaling pathway (Lampard et al., 2008), it will be worthwhile to investigate whether Class III AtOFPs might be phosphorylated by kinases operating downstream of an ER LRR receptor-like kinase signaling pathway, and whether such post-translational modification of OFPs plays a role in regulating silique morphology.

## Author Contributions

SW conceived the review topic, BE and YC participated in the discussion of topic, SW drafted the manuscript, BE reversed the manuscript, and all the authors approved the final version of manuscript.

## Conflict of Interest Statement

The authors declare that the research was conducted in the absence of any commercial or financial relationships that could be construed as a potential conflict of interest.
